# EHMTI-0202. Study protocol of BoTN: a randomized, double-blind, placebo-controlled trial to evaluate the efficacy and safety of botulinum toxin injections in the treatment of trigeminal neuralgia

**DOI:** 10.1186/1129-2377-15-S1-C9

**Published:** 2014-09-18

**Authors:** J Burmeister, D Holle, E Bock, C Ose, HC Diener, M Obermann

**Affiliations:** 1 Department of Neurology, University Hospital Essen, Essen, Germany; 2 Institute for Medical Informatics and Epidemiology, University Hospital Essen, Essen, Germany; 3 Center for Clinical Trials, University Hospital Essen, Essen, Germany

## Background

Trigeminal neuralgia (TN) is a chronic disorder characterized by paroxysmal facial pain. Adequate prophylactic drug therapy is often limited by the lack of efficacy and intolerance due to central nervous system side effects.

Subcutaneous injections of botuinum toxin type A (BT-A) are a promising treatment option for patients with insatisfactory response to drug therapy or neurosurgical intervention.

This is the study protocoll of a prospective, placebo-controlled, double blind clinical trial investigating the add-on therapy of subcutaneous of BT-A injections to standard treatment.

## Methods and design

BoTN is a prospective, randomized, double-blind, placebo-controlled trial with a randomized withdrawal design. Eligible patients with classic TN who are otherwise refractory to medical and neurosurgical treatment will receive subcutaneous injections of BT-A into injection sites of the affected trigeminal branch.

In the first phase phase all patients will receive verum in a single blinded intervention and twelve weeks later therapy responders will be 1:1 allocated to the verum or placebo (saline) arm. This time the intervention is double blind and injections will be done at the same sites.

This trial will be conducted in a tertiary outpatient clinic specialized in the treatment of headache and facial pain.

## Discussion

BoTN is designed to assess the efficacy and safety of local BT-A injections in additon to standard prophylactic treatment.

No conflict of interest.

